# Identification of a pediatric acute hypoxemic respiratory failure signature in peripheral blood leukocytes at 24 hours post-ICU admission with machine learning

**DOI:** 10.3389/fped.2023.1159473

**Published:** 2023-03-17

**Authors:** Jocelyn R. Grunwell, Milad G. Rad, Michael J. Ripple, Nadir Yehya, Hector R. Wong, Rishikesan Kamaleswaran

**Affiliations:** ^1^ Division of Critical Care Medicine, Children’s Healthcare of Atlanta, Atlanta, GA, United States; ^2^ Department of Pediatrics, Emory University School of Medicine, Atlanta, GA, United States; ^3^ Department of Electrical and Computer Engineering, Georgia Institute of Technology, Atlanta, GA, United States; ^4^ Department of Anesthesiology and Critical Care Medicine, University of Pennsylvania, Philadelphia, PA, United States; ^5^ Division of Pediatric Intensive Care Medicine, Children’s Hospital of Philadelphia, Philadelphia, PA, United States; ^6^ Division of Critical Care Medicine, Cincinnati Children’s Hospital Medical Center, Cincinnati, OH, United States; ^7^ Department of Biomedical Informatics, Emory University School of Medicine, Atlanta, GA, United States; ^8^ Department of Biomedical Engineering, Georgia Institute of Technology, Atlanta, GA, United States

**Keywords:** pediatric, acute respiratory distress syndrome, mechanical ventilation, gene expression profiling, machine learning, transcriptomics

## Abstract

**Background:**

There is no generalizable transcriptomics signature of pediatric acute respiratory distress syndrome. Our goal was to identify a whole blood differential gene expression signature for pediatric acute hypoxemic respiratory failure (AHRF) using transcriptomic microarrays within twenty-four hours of diagnosis. We used publicly available human whole-blood gene expression arrays of a Berlin-defined pediatric acute respiratory distress syndrome (GSE147902) cohort and a sepsis-triggered AHRF (GSE66099) cohort within twenty-four hours of diagnosis and compared those children with a P_a_O_2_/F_i_O_2_ < 200 to those with a P_a_O_2_/F_i_O_2_ ≥ 200.

**Results:**

We used stability selection, a bootstrapping method of 100 simulations using logistic regression as a classifier, to select differentially expressed genes associated with a P_a_O_2_/F_i_O_2_ < 200 vs. P_a_O_2_/F_i_O_2_ ≥ 200. The top-ranked genes that contributed to the AHRF signature were selected in each dataset. Genes common to both of the top 1,500 ranked gene lists were selected for pathway analysis. Pathway and network analysis was performed using the Pathway Network Analysis Visualizer (PANEV) and Reactome was used to perform an over-representation gene network analysis of the top-ranked genes common to both cohorts. Changes in metabolic pathways involved in energy balance, fundamental cellular processes such as protein translation, mitochondrial function, oxidative stress, immune signaling, and inflammation are differentially regulated early in pediatric ARDS and sepsis-induced AHRF compared to both healthy controls and to milder acute hypoxemia. Specifically, fundamental pathways related to the severity of hypoxemia emerged and included (1) ribosomal and eukaryotic initiation of factor 2 (eIF2) regulation of protein translation and (2) the nutrient, oxygen, and energy sensing pathway, mTOR, activated *via* PI3K/AKT signaling.

**Conclusions:**

Cellular energetics and metabolic pathways are important mechanisms to consider to further our understanding of the heterogeneity and underlying pathobiology of moderate and severe pediatric acute respiratory distress syndrome. Our findings are hypothesis generating and support the study of metabolic pathways and cellular energetics to understand heterogeneity and underlying pathobiology of moderate and severe acute hypoxemic respiratory failure in children.

## 1. Introduction

Clinical and biological heterogeneity are hallmark features of acute respiratory distress syndrome (ARDS), and correlating clinical phenotypes with biological endotypes using plasma biomarkers and clinical variables using machine learning methods has led to the discovery of a hyperinflammatory (reactive) and hypoinflammatory (unreactive) ARDS endotype with differential responses to high positive end-expiratory pressure (PEEP), low tidal-volume and restrictive fluid therapies (1–4). Systemic infections, such as sepsis, often caused by pneumonia, are a common trigger of ARDS; however, trauma, aspiration, near-drowning, burns, and blood transfusions are also instigators of ARDS.

Statistical and machine learning approaches have been used to perform a meta-analysis of publicly available whole-blood gene expression data to predict mortality from sepsis in adults and children (5). A multi-cohort analysis of adult and pediatric whole-blood gene expression data did not find a generalizable transcriptomics signature of ARDS (6), perhaps due to differences in the analytical approach, the cohort selection, or in the combination of children and adult data. Machine learning random forest models applied to clinically available laboratory and physiologic data within the first two days of intensive care outperformed multivariable logistic regression at predicting prolonged acute hypoxemic respiratory failure (AHRF) of a week of more following influenza infection in a large multicenter cohort of children enrolled in the PICFLU observational study (7). We have used machine learning to explore differentially expressed genes in the primary airway cells from tracheal aspirate samples of mechanically ventilated children with ARDS (8). Since publication of the ARDS transcriptomic meta-analysis incorporating adult ARDS and pediatric sepsis-triggered AHRF (6), pediatric ARDS-specific whole-blood microarray gene expression cohort is now publicly available, thus providing an opportunity to further explore the existence of pediatric ARDS-specific transcriptomic signatures (9).

The P_a_O_2_/F_i_O_2_ ratio is the ratio of arterial oxygen partial pressure (P_a_O_2_ in mmHg) to fractional inspired oxygen (F_i_O_2_ expressed as a fraction, not a percentage). The primary objective of this study was to determine a gene expression signature of mild (P_a_O_2_/F_i_O_2_ ≥ 200) compared with moderate/severe (P_a_O_2_/F_i_O_2_ < 200) AHRF from two publicly available whole blood gene expression microarray datasets. Secondary objectives were to explore the common gene networks of moderate/severe vs. mild AHRF.

## 2. Methods

### 2.1. Ethics approval and consent to participate

Only deidentified clinical data was used in this study. The parent study protocols for collection and use of biological specimens and clinical data were approved by the Institutional Review Board (IRB) at the Children's Hospital of Philadelphia (CHOP IRB 13-010578, “Biomarkers of pediatric ARDS”, Approved 7/2/2014; GSE147902) and by the IRBs of each of the 18 participating institutions for the sepsis AHRF cohort [Cincinnati Children's Hospital Medical Center parent protocol approved by Emory IRB 00079159, “Genomic Analysis of Pediatric Systemic Inflammatory Response Syndrome (SIRS)”, Approved 02/21/2015; GSE66099]. Written informed consent was obtained from legal caregivers prior to enrollment in the parent studies (10–14). Procedures were followed in accordance with the ethical standards of the responsible committee on human experimentation (institutional or regional) and with the Helsinki Declaration of 1975.

### 2.2. Data collection

The pediatric sepsis dataset, GSE66099, and the pediatric ARDS dataset, GSE147902 were downloaded from the NCBI Gene Expression Omnibus (GEO) repository (9, 15) (https://www.ncbi.nlm.nih.gov/geo/). Gene expression data was deidentified and publicly available from the aforementioned two independent observational cohort studies. Both datasets contain gene expression profiles obtained from peripheral whole blood samples of patients who were admitted to the pediatric intensive care unit (PICU) within 24 h of a diagnosis of sepsis or ARDS using the Berlin definition, respectively. Children in GSE66099 were stratified by having a P_a_O_2_/F_i_O_2_ < 200 (vs. ≥200) and defined as having acute hypoxemic respiratory failure as chest radiograph findings were not available to ensure that all ARDS criteria were satisfied. In addition, some children in the GSE66099 cohort did not meet the hypoxemia threshold used in the Berlin definition of ARDS (P_a_O_2_/F_i_O_2_ < 300). By definition, children enrolled in the GSE147902 had to meet al.l Berlin criteria for ARDS (16). For this present analysis, AHRF was defined in both cohorts as a P_a_O_2_/F_i_O_2_ < 200. Pediatric-specific consensus criteria for septic shock and non-pulmonary organ failures were used to determine eligibility for study enrollment (17).

### 2.3. Normalization and background correction

The Affy package from R was used to remove technical variations in the gene expression data (18). Background noise correction and normalizations were performed using the R package gcrma (19). Surrogate Variable Analysis using the R package sva was used to correct for batch effects variation between datasets (20).

### 2.4. Probe to gene mapping

The GSE66099 and GSE147902 microarray dataset Affymetrix probes were matched to gene symbols using Affymetrix Human Genome U133 Plus 2.0 (hgu133plus2.db) and Human Gene 2.1 ST Array (hugene21sttranscriptcluster.db), respectively. Genes with multiple probes were matched to the same gene and the expressions were averaged (5).

### 2.5. Variable selection, classification models, and validation

Graphical modeling or cluster analysis of high-dimensional data [features (*p*) >> number of samples (*n*)]. Stability selection is a “wrapper” algorithm which combines a variety of distinct feature selection algorithms (LASSO, Decision Tree, Recurrent Feature Elimination, etc.) from the Python scikit-learn package to address the nature of this high-dimension data reduction problem (21). This approach is similar to popular “Baruta” R package (22). Stability selection can be seen as the python alternative, which uses subsampling (bootstrap sampling) of the data in combination with a feature selection algorithm such as LASSO to find the most important features in every sampled version of the data. The aggregate results from bootstrap sampling are used to calculate a stability score for each feature in the data. Features were then selected by choosing an appropriate threshold for the stability scores (importance coefficient). We selected candidate markers using stability selection by bootstrapping 100 simulations from all of the data to select stable gene markers using logistic regression as a classifier, with 0.6 as the threshold for selection to ensure enough genes were available for the primary analysis (21). The top-ranked genes that contributed to the AHRF signature were selected in each dataset. Genes common to both of the top 1,500 ranked gene lists were selected for pathway analysis. Leave-one-out cross-validation was used to validate the primary model. Area under the receiver operating characteristic (AUROC) and precision-recall curves were generated using the Python package scikit-learn 1.1.2 (23).

### 2.6. Pathway analysis

**Figure 1:**
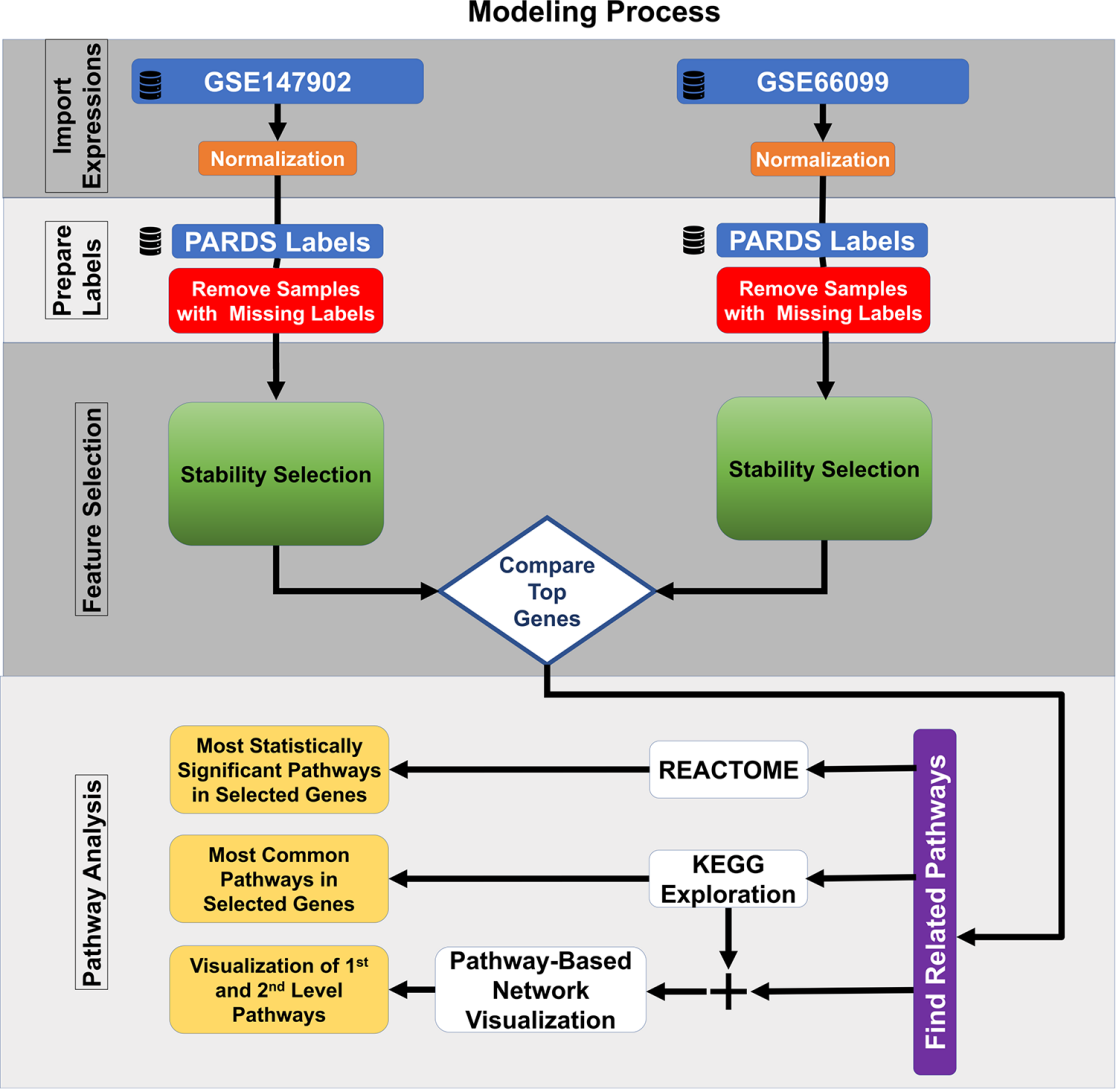
Machine learning feature selection pipeline.

The most common pathways were explored using the 185 top-ranked genes common to both datasets using the Kyoto Encyclopedia of Genes and Genomes (KEGG) (24, 25). The identified KEGG pathways are user defined first-level pathways that were fed into the R package Pathway Network Visualizer (PANEV) to identify and map the second- and third-level pathway networks associated with AHRF (26). The Python Reactome package was used to perform an over-representation gene network analysis using the 185 top-ranked genes common to both datasets (27, 28). We also explored canonical pathway enrichment, upstream regulator, causal network analysis, and molecular and cellular functions with the 185 top-ranked genes common to both datasets using the Ingenuity Pathway Analysis software (IPA, Qiagen, Redwood City, CA). The data analysis workflow using machine learning methods for common dataset gene selection and pathway analysis is shown in Figure 1.

### 2.7. Protein-protein interaction network analysis

We visualized the protein-protein interaction network using the software package Cytoscape 3.2.0 (29). Nodes represent proteins and edges represent interactions between two proteins. Hub genes in the protein-protein interaction network were defined at those with at least a degree of connectivity greater than or equal to ten. These analyses are available in the github repository at https://github.com/ghiasirad/pahrf.git as PPIN.pdf (Supplementary File 5), PPIN.sif (Supplementary File 6), and PPIN.csv (Supplementary File 7) files.

## 3. Results

### 3.1. Cohort description

There were forty-seven healthy control children, sixty-five children without (i.e., P_a_O_2_/F_i_O_2_ ≥ 200) and ninety-six children with (P_a_O_2_/F_i_O_2_ < 200) sepsis-related AHRF from GSE66099 (sepsis dataset). There were twenty-six children without (P_a_O_2_/F_i_O_2_ 201–300) and seventy children with (P_a_O_2_/F_i_O_2_ < 200) AHRF in GSE147902 (pediatric ARDS dataset). The available demographics and clinical characteristics of the participants stratified by P_a_O_2_/F_i_O_2_ ratio are summarized in Table 1.

**Table 1:** Demographic and clinical characteristics of cohort participants.

Characteristics, *n* (%) or median (Q1, Q3)	GSE66099 P_a_O_2_/F_i_O_2_		GSE147902 P_a_O_2_/F_i_O_2_	
	≥200 *n* = 99	<200 *n* = 65	200–299 *n* = 26	<200 *n* = 70
Age (year)	2.4 (1.15, 5.75)	2.4 (0.8, 5.9)	6.7 (0.8, 14.1)	9.4 (2.9, 14.5)
Sex
Female	39 (39.4)	29 (44.6)	7 (27)	31 (44)
Male	60 (60.6)	36 (55.4)	19 (73)	39 (56)
Race/Ethnicity
Black	–^a^	–^a^	4 (15)	26 (37)*
White	–^a^	–^a^	15 (58)	24 (34)*
Hispanic	–^a^	–^a^	6 (23)	8 (11)*
Asian/Pacific Islander	–^a^	–^a^	0 (0)	3 (4.3)*
Other	–^a^	–^a^	1 (3.8)	9 (13)*
Severity of illness
Pediatric Risk of Mortality III	13.0 (9, 19)	16.0 (11, 22)	10 (7, 17)	11 (6, 20)
Non-pulmonary organ failures, *n* (min, max)	2 (1, 6)*	1 (1, 6)	1 (1, 2.75)	1 (1, 2)
Comorbidities
Any comorbidity	43 (44.3)	21 (32.3)	21 (81)	44 (63)
Malignancy	6 (6.2)	3 (4.6)	6 (29)	14 (32)
Immunocompromised	9 (9.3)	7 (10.8)	8 (31)	22 (31)
Bone Marrow or Stem Cell Transplant	4 (4.1)	2 (3.1)	5 (19)	10 (14)
**Clinical Trigger**
Infectious pneumonia	14 (14.1)	17 (26.2)	13 (50)	43 (61)
Non-pulmonary sepsis	85 (85.9)	73 (73.8)	9 (35)	10 (14)
Aspiration pneumonia	NA	NA	1 (3.8)	9 (12.9)
Trauma	NA	NA	0 (0)	3 (4.3)
Other	NA	NA	3 (11.5)	5 (7.1)
Outcomes
PICU mortality	9 (9.1)	13 (20.0)*	8 (31)	12 (17)
Complicated course	16 (16.7)	29 (44.6)*	9 (35)	19 (27)
28-day ventilator-free days	–^a^	–^a^	20 (0, 23)	18 (1, 22)

^a^ Data is not available for reporting.

* Wilcoxon rank sum test; Fisher's exact test; Pearson's *χ*^2^ test, *p* < 0.05.

### 3.2. Selection of common genes of an AHRF signature

Clinically, we were most interested in determining the underlying gene transcripts and molecular pathways that distinguish the most severely hypoxemic and critically ill children with P_a_O_2_/F_i_O_2_ < 200 mmHg from those with milder hypoxemia (P_a_O_2_/F_i_O_2_ ≥ 200 mmHg). We first determined whether the differential gene expression pattern discovered by comparing P_a_O_2_/F_i_O_2_ 200–300 with P_a_O_2_/F_i_O_2_ < 200 from GSE147902 could be applied to a cohort of children with AHRF secondary to sepsis. There was no overlap in differentially expressed genes for children with a P_a_O_2_/F_i_O_2_ < 200 vs. a P_a_O_2_/F_i_O_2_ ≥ 200 using the Benjamini-Hochberg false discovery rate for multiple-hypothesis corrections (30). We therefore used a machine learning stability selection approach to perform this comparison (21, 31).

**Figure 2:**
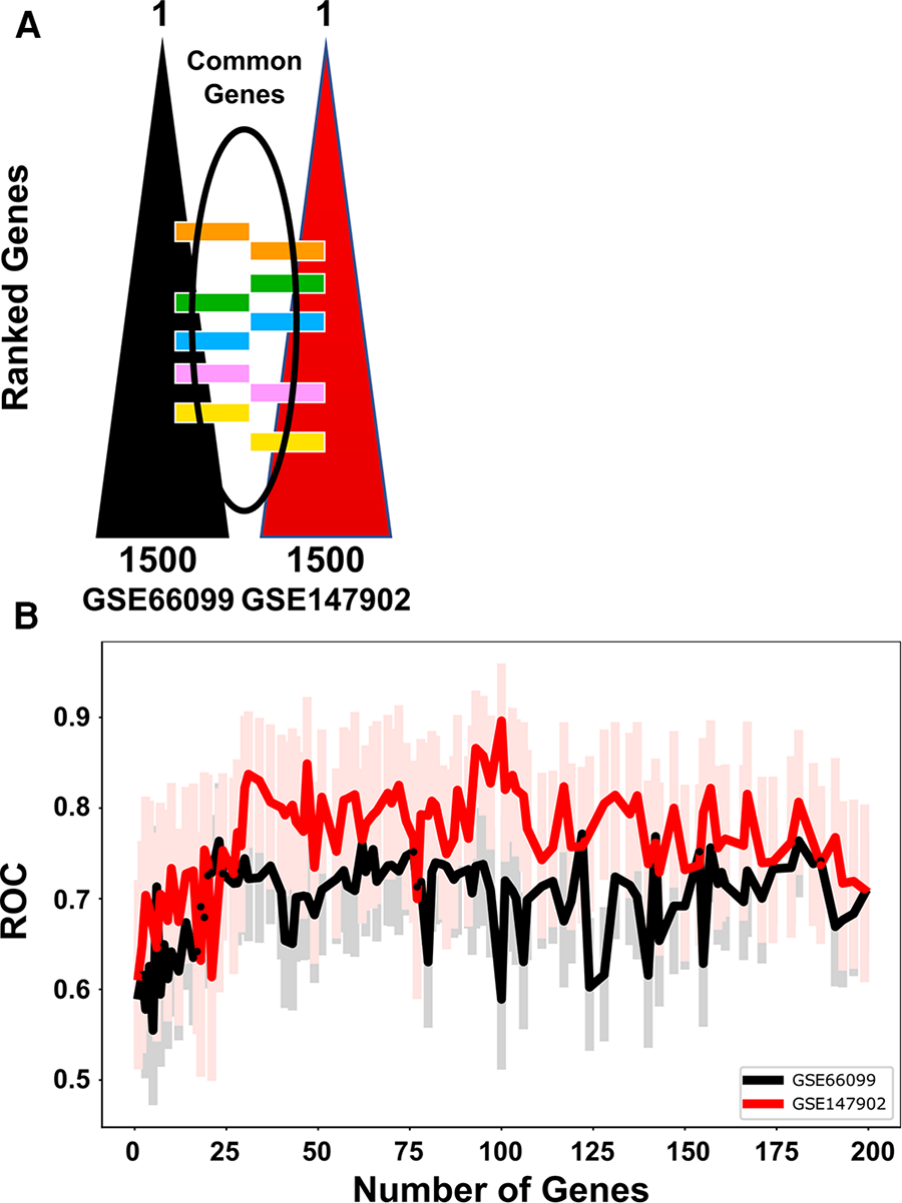
The receiver operative curve (ROC) characteristics for different number of genes (12, 21, 48, 69, 92, 185) selected to model moderate/severe pediatric acute respiratory distress syndrome (ARDS; P_a_O_2_/F_i_O_2_ < 200) vs. mild pediatric ARDS (P_a_O_2_/F_i_O_2_ = 200–300). (**A**) The top 1,500 genes were ordered in a rank-list from GSE66099 and GSE147902. The lists were compared and common genes were added sequentially in the model in groups of 10 genes per iteration. (**B**) The ROC characteristics were calculated for each addition of ten genes in the model up to 185 genes total. The lines show the ROC value for GSE66099 (black) and GSE147902 (red), respectively. The faint red and black lines are the 95% confidence intervals for the ROC for each iteration of the model. The ROC plateaus between 25 and 30 genes for each dataset.

**Figure 3:**
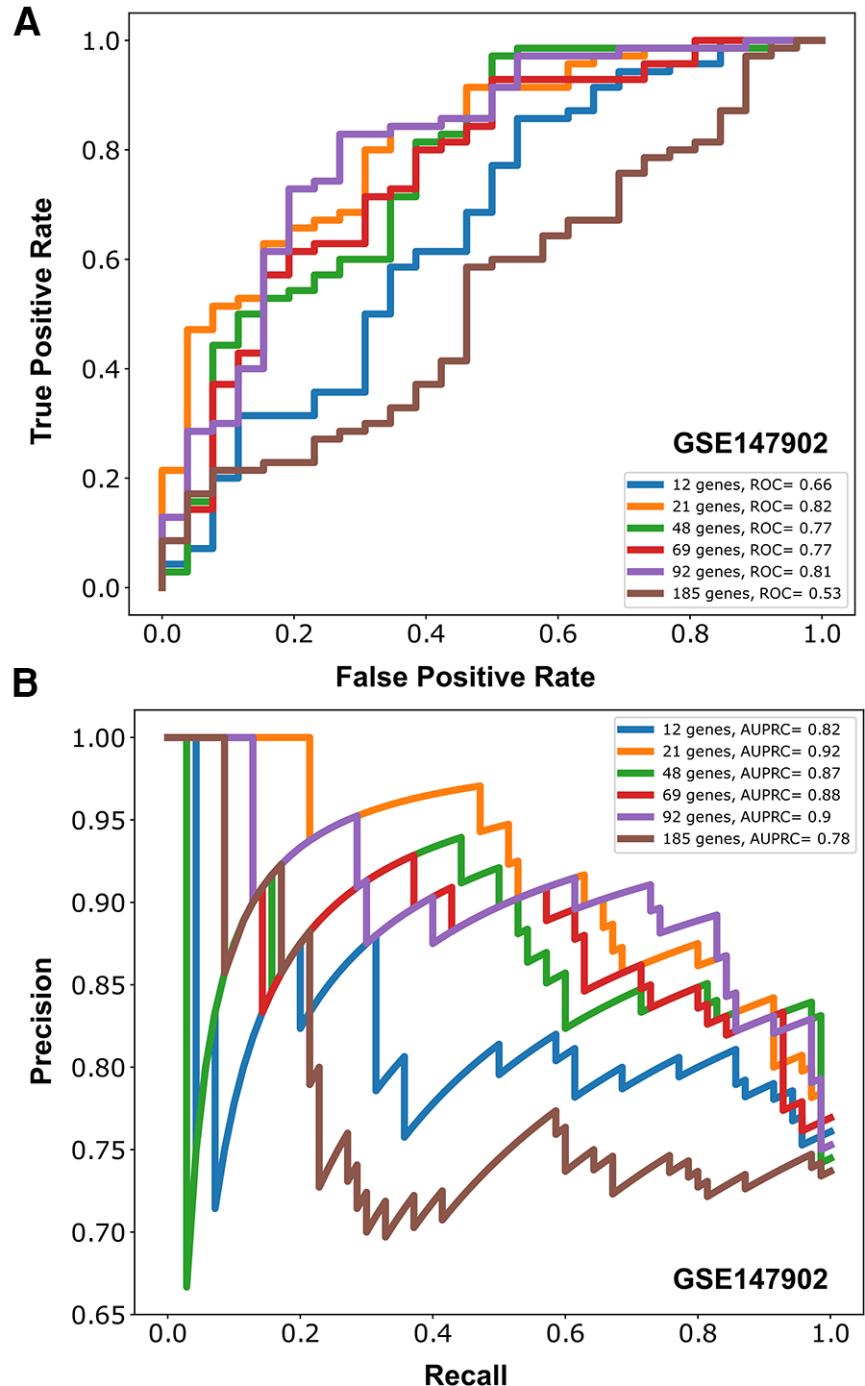
Area under the receiver operative curve (AUROC) (**A**) and area under the precision recall curve (AUPRC) (**B**) for different number of genes (12, 21, 48, 69, 92, 185) selected to model moderate/severe pediatric acute respiratory distress syndrome (ARDS; P_a_O_2_/F_i_O_2_ < 200) vs. mild pediatric ARDS (P_a_O_2_/F_i_O_2_ = 200–300) from GSE147902.

We independently ranked the differentially expressed genes by P_a_O_2_/F_i_O_2_ < 200 vs. P_a_O_2_/F_i_O_2_ ≥ 200 using the machine learning stability select algorithm (21, 31). From the top-ranked 1,500 genes in each data set, genes common to both ranked gene sets were selected in ten gene increments as depicted in Figure 2A. The optimal number of common genes was determined by plotting the area under the receiver operative curve (ROC) characteristic for each incremental addition of ten genes to the AHRF model as shown in Figure 2B. The red line in Figure 2B shows the change in the ROC with the addition of common genes for the pediatric ARDS dataset (GSE147902). The black line in Figure 2B shows the change in the ROC with the addition of common genes for the sepsis dataset (GSE66099). The ROC plateaus around 25–30 genes for each data set. The area under the receiver operating curves (AUROC) and area under the precision recall curves (AUPRC) for GSE147902 (Figures 3A,B) and for GSE66099 (Supplementary Figures S1A,B) are shown for the optimal common genes for each incremental addition of ten genes to the model. For both datasets the AUROC was maximized at 21 genes with a value of 0.82 (95% CI: 0.72–0.89) for GSE147902 and 0.76 (95% CI: 0.69–0.82) for GSE66099 (genes listed in Supplementary Data).

Due to the imbalance in the proportion of cases with and without AHRF in the two cohorts, we report the area under the precision recall curve (AUPRC) for the model of P_a_O_2_/F_i_O_2_ < 200 vs. P_a_O_2_/F_i_O_2_ 201–300. The AUPRC measures the ability of the models to correctly distinguish P_a_O_2_/F_i_O_2_ ≥ 200 from P_a_O_2_/F_i_O_2_ < 200 in both GSE66099 and GSE147902. The pediatric ARDS GSE147902 dataset consists of 27% of children with a P_a_O_2_/F_i_O_2_ ≥ 200 while the sepsis GSE66099 dataset consists of 40% of children with a P_a_O_2_/F_i_O_2_ ≥ 200. The AUPRC was maximized at 92-genes with a value of 0.90 (95% CI: 0.75–0.91) for GSE147902 and 0.66 (95% CI: 0.5–0.76) for GSE66099.

### 3.3. Network analysis

**Figure 4:**
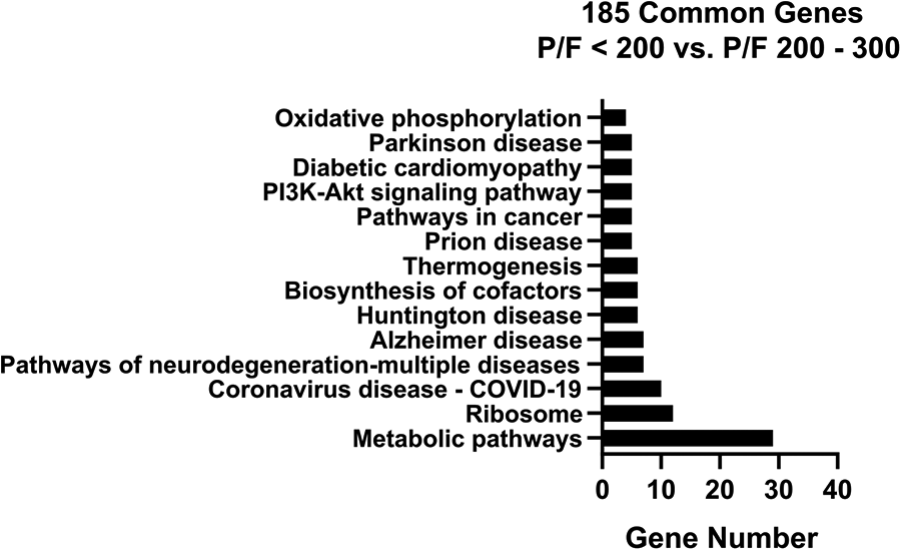
KEGG pathway analysis bar chart with number of genes for the overlapping 185 stability selected ranked genes from GSE147902 and GSE66099 comparing children with a P_a_O_2_/F_i_O_2_ < 200 with a P_a_O_2_/F_i_O_2_ = ≥200.

The 185 genes selected using stability selection method were ranked by normalized importance coefficient for each dataset. The genes retained in the primary model all had importance coefficient scores greater than 0.9. The selected genes, ranked by the number of genes involved in the KEGG pathway, are shown in Figure 4. The top 3 KEGG pathways included metabolic, ribosomal, and coronavirus/COVID-19 disease pathways. Metabolic pathways involve the intricate networks of glycolysis, the tricarboxylic acid (TCA) cycle, the pentose phosphate pathway, gluconeogenesis, fatty acid oxidation and lipid metabolism, one-carbon metabolism, amino acid and cofactor metabolism, and nucleic acid metabolism for the production of energy and substrates for fundamental biological reactions. The coronavirus/COVID-19 disease pathway consists of anti-viral detection *via* the toll-like receptor (TLR) and retinoic acid-inducible gene I (RIG-I) pattern recognition receptor pathways, activation of the NLRP3 inflammasome, and signaling through the pro-inflammatory cytokines IL-6, JAK/STAT, PI3K, and MAPK signaling cascades, and activation of the transcription factor NF-KB. We supported the KEGG pathway analysis using Ingenuity Pathway Analysis (IPA) software, and the top canonical IPA pathways included: the Coronavirus pathogenesis pathway (*p-*value 3.70e-07), Eukaryotic Initiation Factor 2 (eIF2) signaling (*p-*value 4.62e-05), mitochondrial dysfunction (*p-*value 2.95e-04), mammalian target of rapamycin (mTOR) signaling (*p-*value 5.16e-03), and cell cycle control of chromosomal replications (*p-*value 8.6e-03) (Supplementary Table S1). Upstream regulators included La Ribonucleoprotein 1 (*LARP1, p-*value 3.95e-07), Mitogen-Activated Protein Kinase Kinase Kinase Kinase 4 (*MAP4K4, p-*value 4.92e-06), Rapamycin-insensitive companion of mammalian target of rapamycin (*RICTOR, p-*value 9.91e-05), carbohydrate-responsive element-binding protein (*ChREBP*) also known as MLX-interacting protein-like (*MLXIPL, p-*value 2.60e-05), and Brain cytoplasmic 200 long-noncoding RNA (BC200 lncRNA or *BCYRN1, p-*value 1.76e-04). The main molecular and cellular functions included protein synthesis (*p-*value range: 1.49e-02–7.08e-13), RNA Damage and Repair (*p-*value range: 1.48e-06–1.48e-06), Cell Death and Survival (*p-*value range: 2.97e-02–6.06e-05), Cell Morphology (*p-*value range: 2.97e-02–6.06e-05), and Cellular Function and Maintenance (*p-*value range: 2.97e-02–6.06e-05). The top networks were RNA Damage and Repair, Protein Synthesis, RNA Post-Transcriptional Modification (Score 47), Cell Death and Survival, Protein Synthesis, Cancer (Score 44), Protein Synthesis, Cancer, Hematological Disease (Score 20), Skeletal and Muscular System Development and Function, Tissue Morphology, Cell Death and Survival (Score 18), and Cancer, Endocrine System Disorders, Organismal Injury and Abnormalities (Score 16).

We next visualized networks of pathways involved in the moderate/severe AHRF gene expression signature by imputing the list of 185 common genes (Supplementary File 1) into the PANEV package in R. The first-, second-, and third-level pathways with the corresponding genes contributing to each pathway are shown in Supplementary Files 2–5 (Supplementary Files can be found at: https://github.com/ghiasirad/pahrf.git).

Finally, we used the curated Reactome database to perform a pathway over-representation analysis using the 185 common genes defining the moderate/severe AHRF gene expression signature. There were nineteen significantly enriched pathways shown in Table 2 with cytosolic and mitochondrial protein translation, selenoamino acid metabolism, selenocysteine synthesis, and nonsense codon mediated decay of messenger RNA. In addition of gene network analysis, we performed a protein-protein interaction network analysis using 185 top-ranked genes common to both datasets. The protein-protein interaction network analysis showed seventeen proteins with connections to ten or more other proteins (Supplementary Fi1es 6–8).

**Table 2:** Reactome pathways sorted by *p*-value for the top 185 overlapping stability selected ranked genes from GSE147902 and GSE66099 comparing children with a P_a_O_2_/F_i_O_2_ < 200 with a P_a_O_2_/F_i_O_2_ ≥ 200–300.

Pathway name	Entities			Reactions	
	Found	Ratio	FDR^a^	Found	Ratio
Peptide chain elongation	12/134	0.006	8.99e-04	5/5	3.70e-04
Selenoamino acid metabolism	12/198	0.009	0.003	5/33	0.002
Formation of a pool of free 40S subunits	9/111	0.005	0.003	2/2	1.48e-04
Eukaryotic Translation Termination	9/113	0.005	0.003	3/5	3.70e-04
Selenocysteine synthesis	9/115	0.005	0.003	2/7	5.18e-04
SRP-dependent cotranslational protein targeting to membrane	9/119	0.005	0.003	5/5	3.70e-04
Nonsense mediated Decay independent of the Exon Junction Complex	9/121	0.006	0.003	1/1	7.40E-05
Eukaryotic Translational Elongation	12/187	0.009	0.003	8/9	6.66e-04
L13a-mediated translational silencing of Ceruloplasmin expression	9/124	0.006	0.003	2/3	2.22e-04
Viral mRNA Translation	10/135	0.006	0.005	2/2	1.48e-04
Translation	24/755	0.035	0.011	52/99	0.007
GTP hydrolysis and joining of the 60S ribosomal subunit	9/166	0.008	0.018	3/3	2.22e-04
Ribosomal scanning and start codon recognition	6/70	0.003	0.019	2/2	1.48e-04
Mitochondrial translation elongation	8/135	0.006	0.02	5/8	5.92e-04
Mitochondrial translation termination	8/137	0.006	0.02	5/5	3.70e-04
Mitochondrial translation initiation	9/181	0.008	0.025	3/4	2.96e-04
Formation of the ternary complex, and subsequently, the 43S complex	6/86	0.004	0.039	1/3	2.22e-04
Nonsense-Mediated Decay	11/197	0.009	0.039	5/6	4.44e-04
Nonsense Mediated Decay enhanced by the Exon Junction Complex	11/197	0.009	0.039	4/5	3.70e-04

^a^ False Discovery Rate.

We also examined the differential gene expression of pediatric ARDS severity from the pediatric ARDS-specific dataset GSE147902 compared to healthy controls from the GSE66099 dataset. Volcano plots of the mild PARDS vs. healthy controls and moderate/severe PARDS vs. healthy controls are shown in Supplementary Figure S2A,B, respectively. Bar graphs of the number of genes in each KEGG pathway for mild (P_a_O_2_/F_i_O_2_ 200–300 from GSE147902) vs. healthy controls from GSE66099 (Supplementary Figure S3A) and for moderate/severe (P_a_O_2_/F_i_O_2_ < 200 from GSE147902) vs. healthy controls from GSE66099 (Supplementary Figure S3B) are shown. Furthermore, we compared the differential gene expression of moderate/severe (P_a_O_2_/F_i_O_2_ < 200) compared to mild (P_a_O_2_/F_i_O_2_ 200–300) pediatric ARDS in GSE147902 summarized as a bar graph with the number of genes represented in each KEGG pathway (Supplementary Figure S4). The Reactome pathways sorted by *p-*value for the top 200 overlapping stability selected ranked genes from GSE147902 comparing children with a P_a_O_2_/F_i_O_2_ < 200 with a P_a_O_2_/F_i_O_2_ ≥ 200–300 are shown in Supplementary Table S2. These analyses are presented and the findings discussed in the online Supplementary Materials (https://github.com/ghiasirad/pahrf.git).

## 4. Discussion

We used publicly available microarray gene expression data sets and machine learning methods to determine metabolic and immune pathways involved in the systemic response of children within 24 h of an ARDS diagnosis. We evaluated the overlap amongst genes differentiating children with a P_a_O_2_/F_i_O_2_ < 200 vs. a P_a_O_2_/F_i_O_2_ ≥ 200 using a pediatric ARDS-specific cohort and a pediatric sepsis cohort with AHRF. Although the sample collection for these pediatric cohorts predated the COVID-19 pandemic, the Coronavirus pathogenesis pathway was prominent and reflects the systemic inflammatory changes that occur with severe ARDS. Novel pathways including ribosomal and eukaryotic initiation of factor 2 (eIF2) regulation of protein translation and the nutrient, oxygen, and energy sensing pathway, mTOR, which is activated *via* PI3K/AKT signaling, emerged in the KEGG and IPA analysis. We also identified gene networks involved in distinguishing children with mild or moderate/severe pediatric ARDS from healthy controls. Finally, we identified gene networks involved in differentiating children with mild from moderate/severe pediatric ARDS using data from the pediatric ARDS-specific cohort. Our results support further investigation of gene networks and signaling pathways involved in differentiating pediatric ARDS severity and understanding pediatric ARDS heterogeneity by underlying biological processes.

An attempt at finding a generalizable diagnostic gene expression signature for ARDS using publicly available human whole blood gene expression arrays in adults and children failed to find an ARDS-specific signature after adjusting for clinical severity (6). Since this publication, a pediatric-specific ARDS transcriptomics dataset from whole blood was used to identify three ARDS sub-phenotypes with divergent clinical characteristics and outcomes using k-means clustering (9). A limitation of the transcriptomic pediatric ARDS phenotyping study was the lack of a non-ARDS mechanically ventilated cohort as a control group (9). Using the definition of a P_a_O_2_/F_i_O_2_ < 200 for moderate/severe AHRF from GSE66099 and ARDS from GSE147902, we were able to find an overlapping pattern of gene pathways of importance to pediatric ARDS using stability selection modeling and determine the discriminatory ability of the model to predict moderate/severe acute hypoxemia using the intersection of the top 1,500 ranked genes in the pediatric ARDS-specific and pediatric sepsis AHRF cohorts.

Metabolic pathways were the top KEGG pathway in each analysis. Several recent studies have investigated metabolic changes in ARDS (32). The mechanism by which metabolic derangements, such as mitochondrial dysfunction, decreased oxidative phosphorylation, and oxidative stress, lead to bioenergetic failure in ARDS and metabolic reprogramming of the immune system are active areas of investigation. Metabolomics studies of the plasma and airway fluid of patients with ARDS have shown that changes in tricarboxylic acid (TCA) cycle intermediates, such as glucose, alanine and glutamine, are markers of energetic stress on lung epithelial cells (32–38). Microarray analysis of whole-blood gene expression in adults with sepsis-triggered ARDS revealed that the “reactive” or hyperinflammatory subgroup is enriched for genes associated with oxidative phosphorylation, and that this subgroup is also associated with high plasma lactate levels indicative of mitochondrial dysfunction (39).

By contrast, the mitogen-activated protein kinase (MAPK) pathways that control cell proliferation, differentiation, motility, and survival were enriched in the comparatively “uninflamed” subgroup (39). The MAPK pathway is a first-level or second-level pathway found in all of the analyses distinguishing pediatric ARDS from healthy controls and moderate/severe from mild pediatric ARDS. It was recently shown that children with ARDS exhibit similar “reactive/ hyperinflammed” and “uninflamed” phenotypes as shown in adults with ARDS (40); however, this phenotypic classification was not available at the time of accrual of the publicly available gene expression dataset used in this analysis. Therefore, we cannot speculate on the pathobiology underlying class differences (3, 4, 39, 41). The three Children's Hospital of Philadelphia ARDS transcriptomic subtypes (CATS) described by clustering GSE147902 do not conform to the previously described adult phenotypes and are not yet externally validated (9).

Respiratory viral infections are a common trigger of ARDS in children. Pathogenic influenza and coronaviruses use host epigenetic reprogramming to evade the host immune response (42, 43). In the current analysis, histone deacetylases (HDACs) were found to be a top pathway associated moderate/severe ARDS. HDACs regulate chromatin structure and thereby influence gene expression. For example, HDACs repress the production of proinflammatory cytokines in alveolar macrophages in chronic inflammatory lung conditions such as chronic obstructive pulmonary disease (44, 45). HDAC class I and chromatin modifying enzymes were identified as top pathways associated with ARDS mortality in a study using a multi-omics approach with multiple publicly available datasets (46). DNA methylation is another epigenetic modification that emerged in the Reactome analysis that distinguished moderate/severe from mild pediatric ARDS. Alterations in DNA methylation sites in ARDS are related to an imbalance in inflammation, immunity, endothelial and epithelial function, and coagulation (47).

Although children in these cohorts predated the COVID-19 pandemic, we found that gene networks in the KEGG database labeled coronavirus/COVID-19 (hsa05171) were a prominent pathway. This finding is similar to our previous study profiling differentially expressed genes from primary airway cells obtained from tracheal aspirate samples from intubated children with PARDS (8). The COVID-19 pathway includes proinflammatory cytokines such as interleukin (IL)–6, tumor necrosis factor (TNF)–α, nuclear factor kappa-light-chain-enhancer of activated B cells (NF-kB), IL-1β, IL-8, and IL-12 signaling through the Janus kinase (JAK)/signal transducer and activator of transcription (STAT) pathway. Viral pathogen recognition pathways such as Toll-like receptor signaling through MyD88, retinoic acid-inducible gene I (RIG-1)/melanoma differentiation-associated protein 5 pathways, and the antiviral type I interferon (IFN α/β) response and the complement cascade are also highlighted in the COVID-19 gene signaling network.

Machine learning-based analysis of data with class imbalances can be plagued by a number of issues, stemming from the inherent mathematical assumption of equal case to control distributions among many learning algorithms. As a result, these models, when applied in a single train-test instance, may produce random effects that are poorly generalized in external datasets. The traditional AUROC metric poorly characterizes class-based performances, thus necessitating alternative metrics to evaluate the robustness of models. One of the methods to investigate the effects of class imbalance is by the use of metrics such as AUPRC, F1 (harmonic mean of the precision and recall), and bootstrap with replacement. In this work, we use AUPRC and stability selection using bootstrap with replacement to identify a coherent set of “stable” genes that indicate robust predictive performance consistently across 100 bootstrap iterations. We illustrate the AUPRC plot to show strong performance even in the minority class, thus emphasizing the robustness of the learned model.

Our study is limited by the *post hoc* analysis focused on finding a systemic transcriptomic signature of children with moderate/severe ARDS. Only one single-center study of pediatric ARDS exists without a non-hypoxemic respiratory failure control group. We attempted to validate our findings using a multi-center pediatric sepsis-triggered AHRF cohort using the same definitions as in the pediatric ARDS gene array cohort without the bilateral infiltrate radiographic findings and the more stringent requirement of two arterial blood gases at least four hours apart with a P_a_O_2_/F_i_O_2_ < 200. These cohorts capture gene expression differences early in the course of AHRF. The use of a single time point without serial sampling or clinical trajectory information precludes our assessment of temporal changes in gene expression and is a study limitation. As remarked on prior attempts to discover an ARDS transcriptomic signature, whole-blood derived gene expression was used that may be different from the airway-specific transcriptomic response. Concomitant changes in metabolites are not readily available for analysis in these cohorts. Validation of these findings as part of a prospective, multi-center, observational trial enrolling children with PALICC-defined PARDS will be necessary to generalize our results.

## 5. Conclusions

In summary, our analysis demonstrated that changes in gene expression patterns corresponding to metabolic pathways involved in energy balance, fundamental cellular processes such as protein translation, mitochondrial function, oxidative stress, immune signaling, and inflammation are differentially regulated early in pediatric ARDS and sepsis-induced AHRF compared to both healthy controls and to milder, acute hypoxemia. Our findings support the hypothesis that differential regulation of metabolic pathway genes involved in cellular energetics and metabolic pathways are important mechanisms to consider to further our understanding of the heterogeneity and underlying pathobiology of moderate and severe pediatric acute respiratory distress syndrome. Future work should validate the findings from this secondary analysis of publicly available gene expression data using plasma biomarkers in a prospective observational study seeking to associate underlying pathobiology with outcomes in children with ARDS.

## Data Availability

The datasets presented in this study can be found in online repositories. The names of the repository/repositories and accession number(s) can be found in the article/**Supplementary Material**.
