# Supplementary material for: Identification of a pediatric acute hypoxemic respiratory failure signature in peripheral blood leukocytes at 24 hours post-ICU admission with machine learning
Source: Front Pediatr. 2023 Mar 17;11:1159473. doi: 10.3389/fped.2023.1159473 (PMC10063855; doi:10.3389/fped.2023.1159473)
Supplement: Supplementary file 1 [file Datasheet1.pdf]

*Supplementary Material*

# 1 Supplementary Data

## 1.1 Differential Gene Expression of PARDS Severity Compared to Healthy Controls

We determined the differential gene expression profiles for children with PARDS by comparing children with mild ARDS and moderate/severe ARDS from GSE147902 with healthy controls from the GSE66099 data set. We chose GSE147902 as the ARDS data set because children in this cohort met both the Berlin and PALICC criteria to be enrolled. There were 16,221 genes included in the analyses, and there were 4313 genes down-regulated and 1063 genes up-regulated in the mild PARDS vs. healthy controls. There were 4008 genes down-regulated and 1190 genes up-regulated in the moderate/severe PARDS vs. healthy controls. Volcano plots of the mild PARDS vs. healthy controls and moderate/severe PARDS vs. healthy controls are shown in **Supplementary Figure 2A and 2B**, respectively. Pathway analysis was performed after controlling for genes with 95% or more collinearity followed by a stability selection algorithm to select genes by their importance in distinguishing mild or moderate/severe PARDS from controls. Over-representation analysis of differentially expressed genes corresponding to pathways within the Kyoto Encyclopedia of Genes and Genomes (KEGG) database associated with mild and moderate/severe PARDS are shown in **Supplementary Figures 4A** and **4B**, respectively. The top five pathways common, but in a different rank-order, between the mild and moderate/severe PARDS groups include: metabolic pathways, pathways in cancer, Herpes simplex virus 1 infection, Cytokine-cytokine receptors interactions, and the phosphatidylinositol 3-kinase and protein kinase B (PI3K-Akt) signaling pathway.

The PANEV network of first-level and second-level genes and pathways corresponding to mild PARDS vs. healthy controls and to moderate/severe PARDS vs. healthy controls were mapped and are shown in **Supplementary Files 11-16**.

## 1.2 Selection of Differentially Expressed Genes in Moderate/Severe versus Mild PARDS in a PARDS-Specific Cohort (GSE147902)

While the comparison with healthy children was a starting point, we were also interested in the genes that differentiated mild from moderate/severe PARDS. We used a machine learning-based stability selection approach to compare the differences in gene expression between mild vs. moderate/severe PARDS within the GSE147902 group. Selected genes were mapped to KEGG pathways using an over-representation analysis (**Supplementary Figure 5**). Top pathways differentiating mild from moderate/severe PARDS included metabolic pathways, pathways in cancer, pathways of neurodegeneration (multiple diseases). Pathways in cancer encompass many of the individual pathways listed including the cytokine-cytokine receptor interaction, cAMP, PI3K-Akt, MAPK, JAK/STAT, PPARγ signaling, calcium signaling, mTOR, and TGF-β signaling pathways. The neurodegenerative pathway encompasses mitochondrial dysfunction, mitophagy and autophagy, oxidative stress and the formation of reactive oxygen species.

Second- and third-level pathways were discovered using the first-level KEGG pathways and corresponding top 200 genes of importance identified by the stability selection algorithm using PANEV (**Supplementary Files 17-20**). The top three second-level pathways included MAPK signaling, regulation of actin cytoskeleton, and the insulin signaling pathway. The top third-level pathways include natural killer cell mediated cytotoxicity, Parkinson disease, non-

alcoholic fatty liver disease (mediated by TNFα, interleukin-6 (IL-6), insulin, and PPAR), and AMP-activated protein kinase (AMPK) signaling.

We next used the curated Reactome database of pathways and reactions in human biology to perform a pathway over-representation analysis using the 185 common genes defining the moderate/severe vs. mild PARDS transcriptomic signature. There were twenty-three enriched pathways shown in **Supplementary Table 2** with a false discovery rate (FDR) < 0.05. The top pathways found in Reactome included nucleosome assembly, the deacetylation of histones by histone deacetylases (HDACs), RNA Polymerase I promoter opening, the formation of ATP by chemiosmotic coupling, and the formation of a pool of free 40S ribosomal subunits. Fundamental processes such as recognition and removal of damaged DNA bases, methylation of DNA, control of transcription and translation, and dysfunctional pyroptosis leading to cytokine storm were discovered using Reactome.

# 2 Supplementary Figures and Tables

## 2.1 Supplementary Figure 1.

**Supplementary Figure 1.** Area under the receiver operative curve (AUROC) **A)** and area under the precision recall curve (AUPRC) **B)** for different number of genes (12, 21, 48, 69, 92, 185) selected to model moderate/severe pediatric acute respiratory distress syndrome (ARDS; $P_aO_2/F_iO_2$ < 200) versus mild pediatric ARDS $P_aO_2/F_iO_2$ = 200-300 from GSE66099.

## 2.2 Supplementary Figure 2.

**Supplementary Figure 2.** Volcano plots of **A)** mild pediatric acute respiratory distress syndrome (PARDS; $P_aO_2/F_iO_2$ = 200-300) from GSE147902 compared with healthy controls from GSE66099, and **B)** moderate/severe PARDS ($P_aO_2/F_iO_2$ < 200) from GSE147902 compared with healthy controls from GSE66099.

## 2.3 Supplementary Figure 3.

**Supplementary Figure 3.** Bar chart with number of genes represented in each KEGG pathway following differential gene expression analysis of pediatric acute respiratory distress syndrome (PARDS) versus healthy controls followed by stability selection using a collinearity threshold of 0.95. **A)** Mild PARDS ($P_aO_2/F_iO_2$ = 200-300) from GSE147902 compared with healthy controls from GSE66099. **B)** Moderate/Severe PARDS ($P_aO_2/F_iO_2$ < 200) from GSE147902 compared with healthy controls from GSE66099.

## 2.4 Supplementary Figure 4.

**Supplementary Figure 4.** Bar chart with number of genes represented in each KEGG pathway following stability selection of moderate/severe pediatric acute respiratory distress syndrome (PARDS; $P_aO_2/F_iO_2$ < 200) versus mild PARDS ($P_aO_2/F_iO_2$ = 200-300) from GSE147902.

**2.5 Supplementary Table 1.** Ingenuity Pathway Analysis of the top canonical pathways, upstream regulators, causal network, and molecular and cellular functions for genes for the overlapping 185 stability selected ranked genes from GSE147902 and GSE66099 comparing children with a $P_aO_2/F_iO_2 < 200$ with a $P_aO_2/F_iO_2 = \geq 200$.

**2.6 Supplementary Table 2.** Reactome pathways sorted by p-value for the top 200 overlapping stability selected ranked genes from GSE147902 comparing children with a $P_aO_2/F_iO_2 < 200$ with a $P_aO_2/F_iO_2 \geq 200 - 300$.

**2.7 Supplementary Table 3.** Protein-protein interaction network analysis using the top 185 overlapping stability selected ranked genes from GSE147902 and GSE 66099 comparing children with a $P_aO_2/F_iO_2 < 200$ with a $P_aO_2/F_iO_2 \geq 200$.

## 3 Supplementary Files.

Supplementary Files can be found at: https://github.com/ghiasirad/pahrf.git

**3.1 Supplementary File 1.** List of the 185 genes in common to GSE66099 and GSE 147902.

**3.2 Supplementary File 2.** PANEV first-level (1L) gene list for the overlap of stability selection ranked genes from GSE 147902 and GSE66099 comparing children with a $P_aO_2/F_iO_2 < 200$ with a $P_aO_2/F_iO_2 \geq 200$.

**3.3 Supplementary File 3.** PANEV second-level (2L) gene list for the overlap of stability selection ranked genes from GSE 147902 and GSE66099 comparing children with a $P_aO_2/F_iO_2 < 200$ with a $P_aO_2/F_iO_2 \geq 200$.

**3.4 Supplementary File 4.** PANEV second-level (3L) gene list for the overlap of stability selection ranked genes from GSE 147902 and GSE66099 comparing children with a $P_aO_2/F_iO_2 < 200$ with a $P_aO_2/F_iO_2 \geq 200$.

**3.5 Supplementary File 5.** PANEV network (first-, second-, and third-level) of the common stability selection ranked genes from GSE 147902 and GSE66099 comparing children with a $P_aO_2/F_iO_2 < 200$ with a $P_aO_2/F_iO_2 \geq 200$.

**3.6 Supplementary File 6.** Cytoscape protein-protein interaction network visualization of the 185 top-ranked genes common to the GSE 147902 and GSE66099 datasets comparing children with a $P_aO_2/F_iO_2 < 200$ with a $P_aO_2/F_iO_2 \geq 200$. This file is labeled "PPIN.pdf".

**3.7 Supplementary File 7.** Cytoscape protein-protein interaction network visualization of the 185 top-ranked genes common to the GSE 147902 and GSE66099 datasets comparing children with a $P_aO_2/F_iO_2 < 200$ with a $P_aO_2/F_iO_2 \geq 200$. This file is labeled "PPIN.sif".

**3.8 Supplementary File 8.** Cytoscape protein-protein interaction network visualization of the 185 top-ranked genes common to the GSE 147902 and GSE66099 datasets comparing children with a $P_aO_2/F_iO_2 < 200$ with a $P_aO_2/F_iO_2 \geq 200$. This file is labeled "PPIN.csv".

**3.9 Supplementary File 9.** Differentially expressed genes for mild pediatric acute respiratory distress syndrome (ARDS; $P_aO_2/F_iO_2 = 200\text{-}300$) from GSE147902 compared with healthy controls from GSE66099.

**3.10 Supplementary File 10.** Differentially expressed genes for moderate/severe ARDS ($P_aO_2/F_iO_2 < 200$) from GSE147902 compared with healthy controls from GSE66099.

**3.11 Supplementary File 11.** PANEV first-level (1L) gene list for mild pediatric acute respiratory distress syndrome (ARDS; $P_aO_2/F_iO_2 = 200\text{-}300$) from GSE147902 compared with healthy controls from GSE66099.

**3.12 Supplementary File 12.** PANEV second-level (2L) gene list for mild pediatric acute respiratory distress syndrome (ARDS; $P_aO_2/F_iO_2 = 200\text{-}300$) from GSE147902 compared with healthy controls from GSE66099.

**3.13 Supplementary File 13.** Summary of genes corresponding to the second-level (2L) PANEV pathways for mild pediatric acute respiratory distress syndrome (ARDS;

$P_aO_2/F_iO_2$ = 200-300) from GSE147902 compared with healthy controls from GSE66099.

3.14 **Supplementary File 14.** PANEV first-level (1L) gene list for moderate/severe ARDS ($P_aO_2/F_iO_2$ < 200) from GSE147902 compared with healthy controls from GSE66099.

3.15 **Supplementary File 15.** PANEV second-level (2L) gene list for moderate/severe ARDS ($P_aO_2/F_iO_2$ < 200) from GSE147902 compared with healthy controls from GSE66099.

3.16 **Supplementary File 16.** Summary of genes corresponding to the second-level (2L) PANEV pathways for moderate/severe ARDS ($P_aO_2/F_iO_2$ < 200) from GSE147902 compared with healthy controls from GSE66099.

3.17 **Supplementary File 17.** PANEV first-level (1L) gene list for moderate/severe pediatric acute respiratory distress syndrome (ARDS; $P_aO_2/F_iO_2$ < 200) versus mild ARDS ($P_aO_2/F_iO_2$ = 200-300) from GSE147902.

3.18 **Supplementary File 18.** PANEV second-level (2L) gene list for moderate/severe pediatric acute respiratory distress syndrome (ARDS; $P_aO_2/F_iO_2$ < 200) versus mild ARDS ($P_aO_2/F_iO_2$ = 200-300) from GSE147902.

3.19 **Supplementary File 19.** PANEV third-level (3L) gene list for moderate/severe pediatric acute respiratory distress syndrome (ARDS; $P_aO_2/F_iO_2$ < 200) versus mild ARDS ($P_aO_2/F_iO_2$ = 200-300) from GSE147902.

3.20 **Supplementary File 20.** PANEV network (first-, second-, and third-level) for moderate/severe pediatric acute respiratory distress syndrome (ARDS; $P_aO_2/F_iO_2$ < 200) versus mild ARDS ($P_aO_2/F_iO_2$ = 200-300) from GSE147902.

**Supplementary Figure 1.**

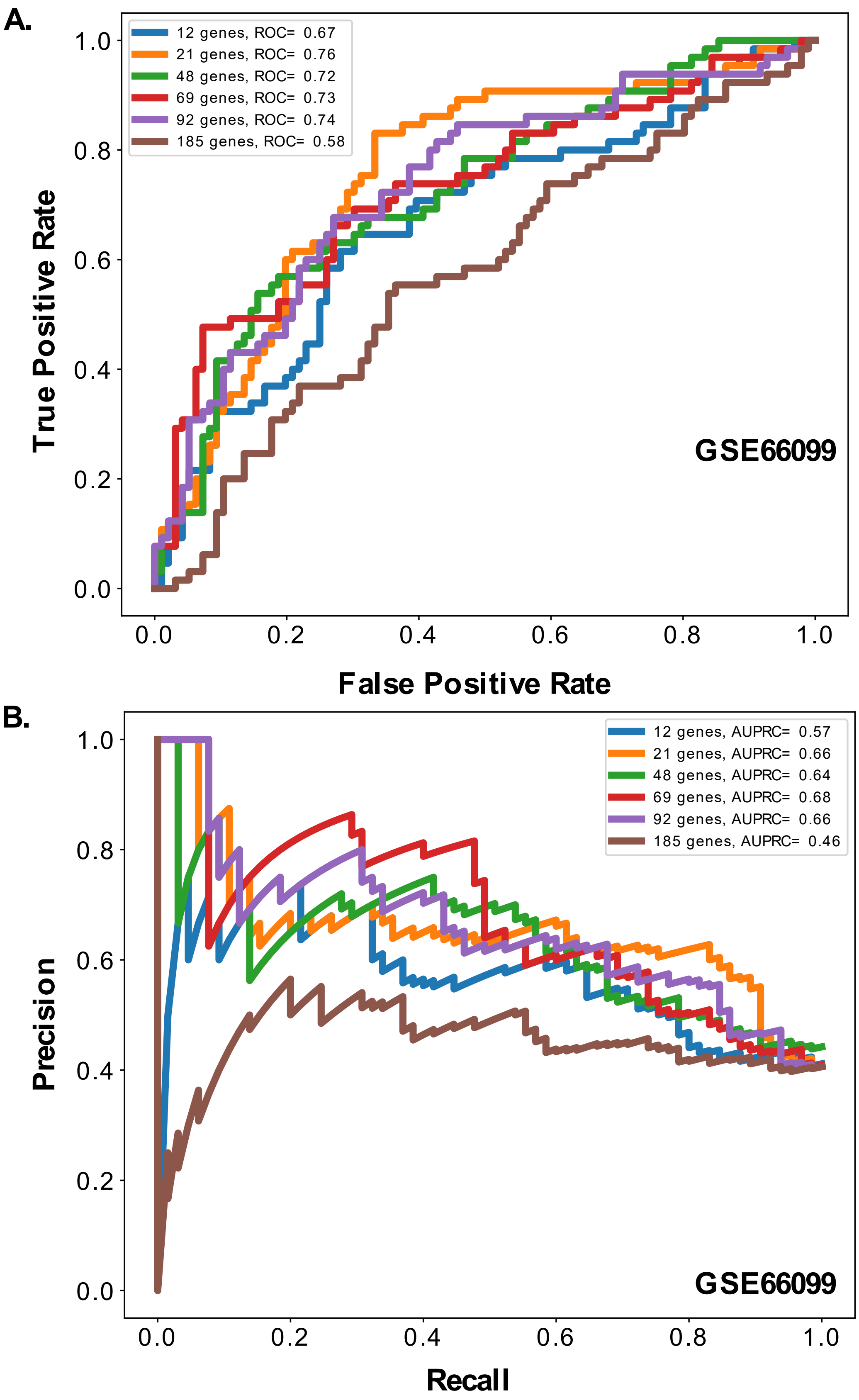

**Supplementary Figure 2.**

**A.**

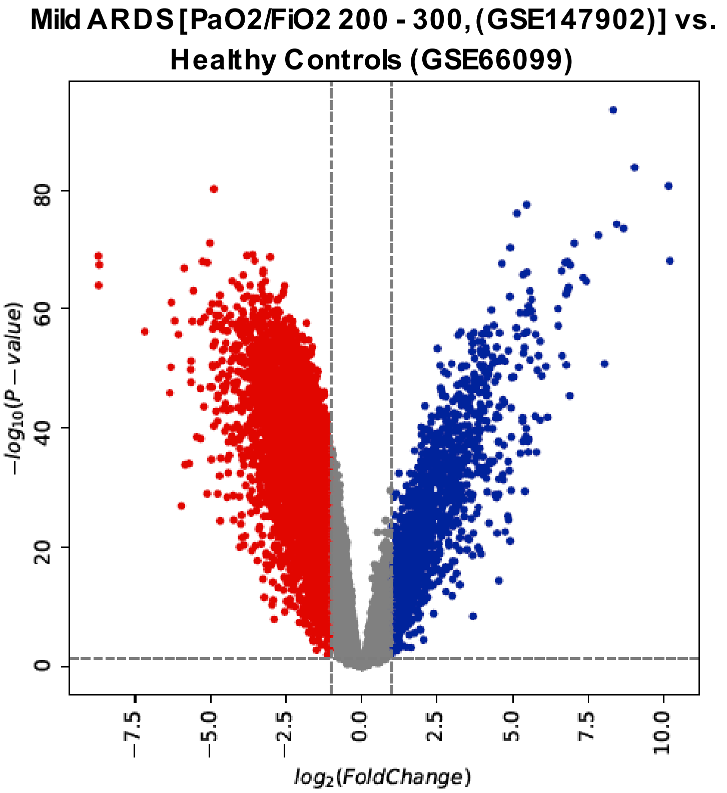


**B.**

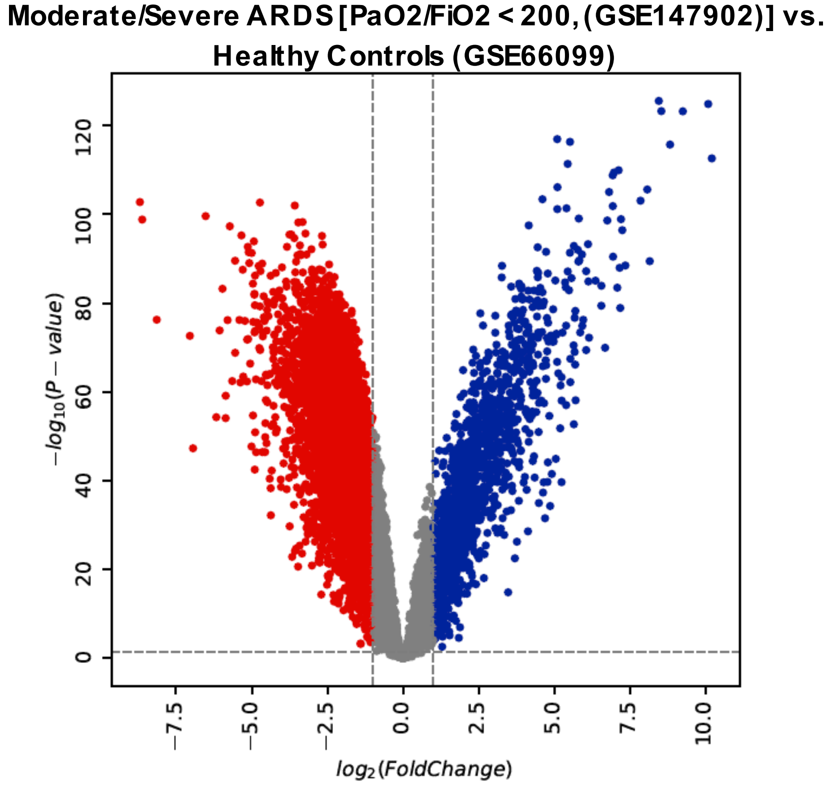

**Supplementary Figure 3.**

A.

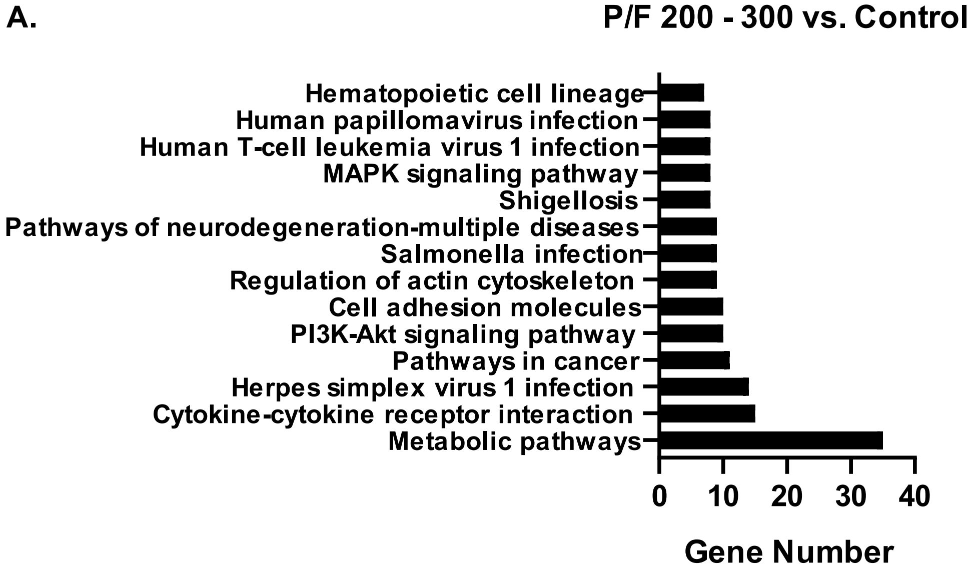


B.

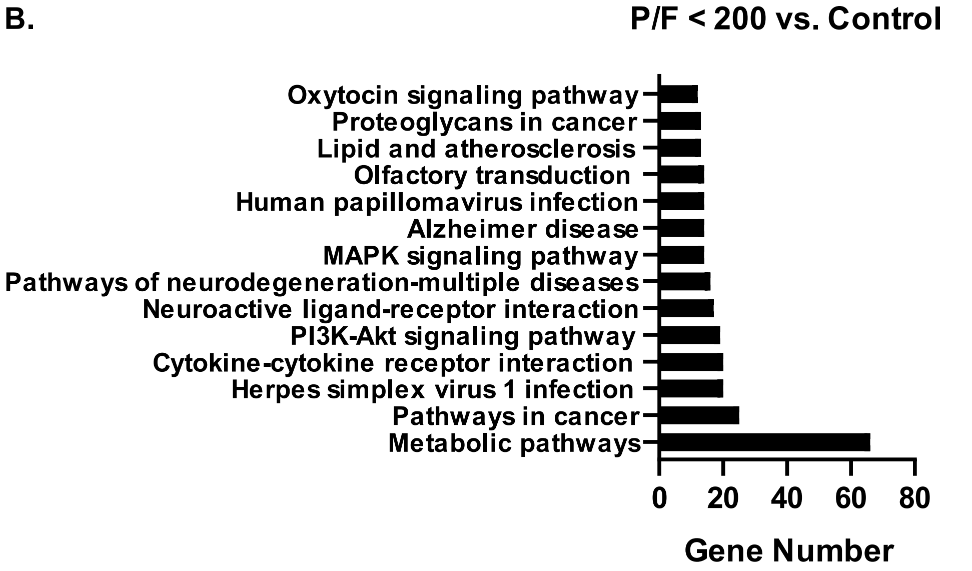

**Supplementary Figure 4.**

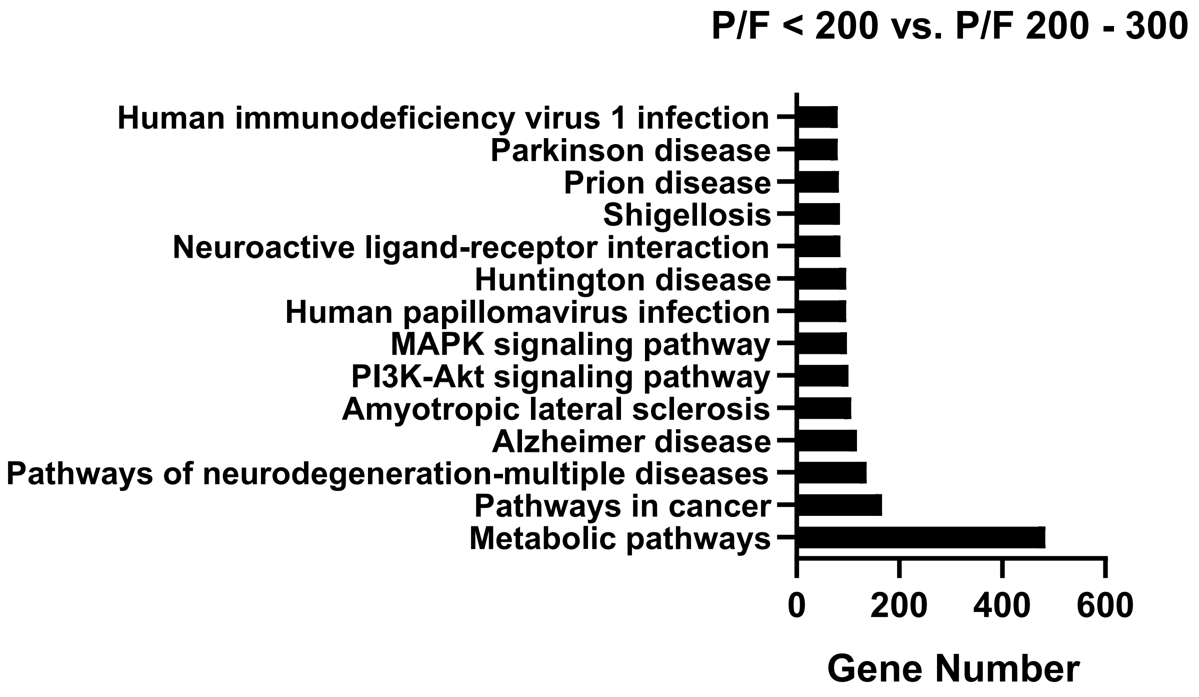

**Supplementary Table 1. Ingenuity Pathway Analysis of the overlapping 185 stability selected ranked genes from GSE147902 and GSE66099 comparing children with a $PaO_2/F_iO_2 < 200$ with a $PaO_2/F_iO_2 \geq 200$.**

| Pathway name | *p*-value | Overlap |
|---|---|---|
| **Top Canonical Pathways** | | |
| Coronavirus Pathogenesis Pathway | 3.70e-07 | 5.5% (11/201) |
| EIF2 Signaling | 4.62e-05 | 4.1% (9/221) |
| Mitochondrial Dysfunction | 2.95e-04 | 4.1% (7/169) |
| mTOR Signaling | 5.16e-03 | 2.9% (6/210) |
| Cell Cycle Control of Chromosomal Replication | 8.6e-03 | 5.4% (3/56) |
| **Upstream Regulators** | | |
| LARP1 | 3.95e-07 | |
| MAP4K4 | 4.92e-06 | |
| RICTOR | 9.91e-05 | |
| MLXIPL | 1.50e-04 | |
| BCYRN1 | 1.76e-04 | |
| **Causal Network** | | |
| LARP1 | 3.59e-07 | |
| MAP4K4 | 4.92e-06 | |
| MLXIPL | 2.60e-05 | |
| MAD1L1 | 5.49e-05 | |
| JUNB | 6.68e-05 | |
| **Molecular and Cellular Functions** | ***p*-value range** | **# Molecules** |
| Protein Synthesis | 1.49e-02 – 7.08e-13 | 35 |
| RNA Damage and Repair | 1.48e-06 – 1.48e-06 | 8 |
| Cell Death and Survival | 2.97e-02 – 6.06e-05 | 18 |
| Cell Morphology | 2.97e-02 – 6.06e-05 | 20 |
| Cellular Function and Maintenance | 2.97e-02 – 6.06e-05 | 34 |
| **Top Networks** | | **Score** |
| RNA Damage and Repair, Protein Synthesis, RNA Post-Transcriptional Modification……………………………………………………………………….. | | 47 |
| Cell Death and Survival, Protein Synthesis, Cancer……………………………. | | 44 |
| Protein Synthesis, Cancer, Hematological Disease…………………………….. | | 20 |
| Skeletal and Muscular System Development and Function, Tissue Morphology, Cell Death and Survival…………………………………………….. | | 18 |
| Cancer, Endocrine System Disorders, Organismal Injury and Abnormalities… | | 16 |

**Supplementary Table 2. Reactome pathways sorted by p-value for the top 200 overlapping stability selected ranked genes from GSE147902 comparing children with a $PaO_2/F_iO_2$ < 200 with a $PaO_2/F_iO_2$ ≥ 200 - 300.**

| Pathway name | Entities Found | Entities Ratio | FDR* | Reactions Found | Reactions Ratio |
|---|---|---|---|---|---|
| Nucleosome assembly | 8 / 54 | 0.002 | 3.55e-07 | 4 / 4 | 2.96e-04 |
| Deposition of new CEPNA-containing nucleosomes at the centromere | 8 / 54 | 0.002 | 3.55e-04 | 4 / 4 | 2.96e-04 |
| HDACs deacetylate histones | 8 / 73 | 0.003 | 0.002 | 5 / 5 | 3.70e-04 |
| RNA Polymerase I Promoter Opening | 6 / 38 | 0.002 | 0.003 | 1 / 2 | 1.48e-04 |
| Formation of ATP by chemiosmotic coupling | 5 / 23 | 0.001 | 0.003 | 3 / 3 | 2.22e-04 |
| Formation of a pool of free 40S subunits | 9 / 111 | 0.005 | 0.003 | 2 / 2 | 1.48e-04 |
| Recognition and association of DNA glycosylase with site containing an affected purine | 6 / 41 | 0.002 | 0.003 | 2 / 10 | 7.40e-04 |
| Cleavage of the damaged purine | 6 / 45 | 0.002 | 0.004 | 2 / 9 | 6.66e-04 |
| L13a-mediated translational silencing of Ceruloplasmin expression | 9 / 124 | 0.006 | 0.004 | 2 / 3 | 2.22e-04 |
| PRC2 methylates histones and DNA | 6 / 48 | 0.002 | 0.004 | 4 / 4 | 2.96e-04 |
| Depurination | 6 / 48 | 0.002 | 0.004 | 4 / 19 | 0.001 |
| Cleavage of the damaged pyrimidine | 6 / 51 | 0.002 | 0.006 | 1 / 20 | 0.001 |
| Defective pyroptosis | 6 / 56 | 0.003 | 0.008 | 1 / 3 | 2.22e-04 |
| DNA methylation | 6 / 60 | 0.003 | 0.012 | 7 / 7 | 5.18e-04 |
| RNA Polymerase I Promoter Escape | 6 / 64 | 0.003 | 0.015 | 1 / 2 | 1.48e-04 |
| Ribosomal scanning and start codon recognition | 6 / 70 | 0.003 | 0.022 | 2 / 2 | 1.48e-04 |
| GTP hydrolysis and joining of the 60S ribosomal subunit | 11/ 166 | 0.008 | 0.022 | 3 / 3 | 2.22e-04 |
| Packaging of telomere ends | 6 / 71 | 0.003 | 0.022 | 2 / 2 | 1.48e-04 |
| Recognition and associated of DNA glycosylase with site containing as affected pyrimidine | 6/ 76 | 0.004 | 0.03 | 1 / 21 | 0.002 |
| Selenocysteine synthesis | 7/115 | 0.005 | 0.044 | 3 / 7 | 5.18e-04 |
| Depyrimidination | 6 / 85 | 0.004 | 0.047 | 2 / 41 | 0.003 |
| Formation of the ternary complex, and subsequently, the 43S complex | 6 / 86 | 0.004 | 0.047 | 1 / 3 | 2.22e-04 |
| SRP-dependent cotranslational protein targeting to membrane | 7/ 119 | 0.005 | 0.047 | 5 /5 | 3.70e-04 |

*False Discovery Rate

**Supplementary Table 3. Protein-protein interaction network analysis of overlapping stability selected ranked genes from GSE147902 and GSE66099 comparing children with a $PaO_2/F_iO_2$ < 200 with a $PaO_2/F_iO_2$ ≥ 200 - 300.**

| Protein name | Abbreviation | Degree (Connections) |
|---|---|---|
| Bifunctional glutamate/proline--tRNA ligase | EPRS | 22 |
| Large ribosomal subunit protein uL10 | RPLP0 | 22 |
| Small ribosomal subunit protein uS13 | RPS18 | 19 |
| Small ribosomal subunit protein uS4 | RPS9 | 18 |
| Mitochondrial ribosomal protein S15 | MRPS15 | 17 |
| Large ribosomal subunit protein eL27 | RPL27 | 16 |
| Small ribosomal subunit protein eS8 | RPS8 | 16 |
| Cell growth-inhibiting gene 33 protein | RPL35A | 16 |
| Small ribosomal subunit protein eS28 | RPS28 | 16 |
| Translation initiation factor IF-2, mitochondrial | MTIF2 | 15 |
| Eukaryotic translation elongation factor 1 alpha 1 | EEF1A1 | 15 |
| c-Myc-binding protein Mm-1 | PFDN5 | 14 |
| Small ribosomal subunit protein eS27 | RPS27 | 13 |
| Mitochondrial ribosomal protein L9 | MRPL9 | 13 |
| Mitochondrial ribosomal protein L33 | MRPL33 | 12 |
| ATP synthase, H+ transporting, mitochondrial Fo complex, subunit d | ATP5H | 11 |
| Mitochondrial ribosomal protein L47 | MRPL47 | 10 |

**List of the 21 Genes in the Model comparing children with $P_aO_2/F_iO_2 < 200$ vs. $P_aO_2/F_iO_2 \geq 200$**

1. LOC100130987
2. SLC25A33
3. NDUFA6
4. ANAPC5
5. ATP5PO
6. MRPL33
7. FASLG
8. MAP4K3
9. PHYH
10. APH1A
11. NEK8
12. ATP8A2
13. NME3
14. ETHE1
15. COMMD9
16. MTO1
17. LY86
18. RPL27
19. DHX57
20. SLC25A47
21. CPVL

frontiers
